# Identification of key genes modules linking diabetic retinopathy and circadian rhythm

**DOI:** 10.3389/fimmu.2023.1260350

**Published:** 2023-12-06

**Authors:** Feng Ling, Caijie Zhang, Xin Zhao, Xiangyang Xin, Shaozhen Zhao

**Affiliations:** ^1^ Tianjin Key Laboratory of Retinal Functions and Diseases, Tianjin Branch of National Clinical Research Center for Ocular Disease, Eye Institute and School of Optometry, Tianjin Medical University Eye Hospital, Tianjin, China; ^2^ Department of Ophthalmology, Inner Mongolia Baogang Hospita, Inner Mongolia, Baotou, China

**Keywords:** diabetic retinopathy, circadian rhythms, biological clock, macrophages, drug screening

## Abstract

**Background:**

Diabetic retinopathy (DR) is a leading cause of vision loss worldwide. Recent studies highlighted the crucial impact of circadian rhythms (CR) on normal retinopathy in response to the external light cues. However, the role of circadian rhythms in DR pathogenesis and potential investigational drugs remains unclear.

**Methods:**

To investigate the weather CR affects DR, differential expression analysis was employed to identify differentially expressed genes (DEGs) from the GEO database (GSE160306). Functional enrichment analysis was conducted to identify relevant signaling pathways. LASSO regression was utilized to screen pivotal genes. Weighted gene co-expression network anlaysis (WGCNA) was applied to identify different modules. Additionally, we use the Comparative Toxicogenomics Database (CTD) database to search key genes related to drugs or molecular compounds. The diabetic mouse model received three consecutive intraperitoneal injections of streptozotocin (STZ) during 3 successive days.

**Results:**

We initially identified six key genes associated with circadian rhythm in DR, including *COL6A3*, *IGFBP2*, *IGHG4*, *KLHDC7A*, *RPL26P30*, and *MYL6P4*. Compared to normal tissue, the expression levels of COL6A3 and IGFB2 were significantly increased in DR model. Furthermore, we identified several signaling pathways, including death domain binding, insulin-like growth factor I binding, and proteasome binding. We also observed that COL6A3 was positively correlated with macrophages (cor=0.628296895, p=9.96E-08) and Th17 cells (cor=0.665120835, p=9.14E-09), while IGFBP2 showed a negatively correlated with Tgd (cor=-0.459953045, p=0.000247284) and Th2 cells (cor=-0.442269719, p=0.000452875). Finally, we identified four drugs associated with key genes: Resveratrol, Vitamin E, Streptozocin, and Sulindac.

**Conclusion:**

Our findings revealed several key genes related to circadian rhythms and several relevant drugs in DR, providing a novel insight into the mechanism of DR and potential implications for future DR treatment. This study contributes to a better understanding of CR in DR and its implications for future therapeutic interventions.

## 1 Introduction

Diabetic retinopathy (DR) stands as a significant global cause of vision loss and represents one of the most prevalent chronic complications associated with diabetes (1). It is characterized by a series of distinct retinal microvascular lesions induced by diabetes, ultimately leading to visual impairment and potential blindness (2). The manifestations of DR encompass microangioma, exudation, neovascularization, retinal detachment, and in severe cases, complete loss of vision (3–5). As the global prevalence of diabetes continues to escalate, there is a corresponding surge in the incidence of DR (5). According to the International Diabetes Federation (IDF), there were 382 million reported cases of diabetes worldwide in 2013, with China bearing the highest burden of diabetic patients aged 20-79, totaling 9.8 million individuals (6). Diabetic retinopathy has emerged as the primary cause of preventable blindness among adults in numerous countries. The severity of diabetic retinopathy can be categorized into two distinct classes: non-proliferative diabetic retinopathy (NPDR) and proliferative diabetic retinopathy (PDR) (7). NPDR represents the most prevalent manifestation of diabetic retinopathy. Initially, it is characterized by features such as edema and the presence of hard exudates, which are essentially lipids leaked from abnormal blood vessels, primarily affecting the central retina, and culminating in blurred central vision (8, 9). In the later stages, PDR is distinguished by the formation of anomalous blood vessels and the development of scar tissue on the retinal surface, subsequently establishing firm adhesions with the posterior vitreous surface (10). This adhesion leads to traction by the vitreous, potentially resulting in hemorrhages from the blood vessels into the vitreous cavity. Ling et al. identified differential DNA methylation of 349 CpG sites representing 233 unique genes in cases with PDR (11). Epigenetic mechanisms are expected to be involved in the pathogenesis of PDR as well (12). NPDR may develop into PDR, where hallmarks of neovascularization of the retina and vitreous hemorrhage are found. The primary treatment approach for NPDR typically revolves around laser photocoagulation targeting macular edema, whereas managing recurrent hemorrhages features prominently in the treatment of PDR. The intricate molecular mechanisms governing the pathogenesis and progression of DR remain largely elusive. To identify novel pivotal genes that are involved in the etiology and progression of DR could be helpful to the research and treatment of DR.

While the pathogenesis of DR is complex and multifactorial, emerging evidence suggests a compelling association between circadian rhythm disruption and the development of DR (13–15). Circadian rhythms, characterized by biological oscillations synchronized with the 24-hour day-night cycle, play a crucial role in regulating various biological and pathological processes (16). These rhythms are synchronized with external light cues, enabling organisms to adapt to the daily environmental changes (17, 18). Emerging evidence has implicated circadian rhythm disturbances in tumorigenesis and several other diseases. Notably, the involvement of circadian rhythm in the etiology of colorectal cancer (16), prostate cancer (19), and bladder cancer (20) has been well-documented. Furthermore, accumulating evidence highlights the significant role of circadian rhythm in modulating the cellular microenvironment (21–23). The eye as a light-sensing organ plays a key role in regulating the circadian rhythms. Consequently, the disruption of circadian rhythm has profound effects on normal ocular regulation. Circadian rhythm dysfunction at multiple levels has been implicated in the pathogenesis of DR (24). For instance, the retinal neuropil is known to express the classic clock genes (e.g., *Bmal*, *Clock*, *Per1*), and retinal circadian system plays a vital role in a range of physiological functions of the eye, like visual processing, disc shedding and phagocytosis, and susceptibility to light-induced photoreceptor damage (17, 25). Silencing of retinal Bmal, for instance, has been shown to significantly decrease cone viability, lead to abnormalities in rod bipolar cells, and thinning of the outer plexiform layer in aged mice (26). Additionally, the knockout of *Per1* and *Per2* genes in the retina has been found to downregulate blue cone opsin and impact its spatial distribution during early postnatal development and throughout maturation (27). Considering the significant influence of the microenvironment on the etiology and progression of various diseases, investigating the intricate relationship between circadian rhythm and the microenvironment holds great potential for advancing our understanding and improving treatment strategies for diabetic retinopathy.

Circadian rhythm dysfunction manifests in diverse facets of DR, ranging from diurnal variations in retinal structure to cellular dysfunction and metabolic imbalances (17, 28). Intriguingly, a rat model of type 2 diabetes exhibited downregulation of a diverse array of clock genes (29). Perturbations in circadian rhythm can profoundly impact the retinal microenvironment, inciting chronic low-grade inflammation and oxidative stress, both cardinal features of DR. Aberrant expression of clock genes within retinal cells regulates the release of pro-inflammatory cytokines, chemokines, and reactive oxygen species (30). These molecular aberrations contribute to the activation of inflammatory pathways and the ensuing impairment of the blood-retinal barrier, thereby facilitating immune cell infiltration into retinal tissue and amplifying retinal damage. Furthermore, circadian rhythm disturbances extend their reach to metabolism and energy homeostasis. For instance, disrupted circadian rhythmicity has been associated with perturbed glucose metabolism and compromised mitochondrial function (31, 32). Nonetheless, the specific impact of circadian rhythm disruption on metabolic disorders in the context of diabetic retinopathy still eludes us, despite the well-established metabolic foundation of this ocular ailment. Comprehending the intricate relationship between circadian rhythm disturbances and the progression of diabetic retinopathy holds immense potential in illuminating novel therapeutic avenues for addressing this debilitating disorder with a propensity for sight loss.

Considering the extensive influence of circadian rhythm disorder in cancers and cardiovascular disease, we speculated that circadian rhythm could also exert an important effect on the development of DR. Consequently, the principal objective of this study was to employ bioinformatics analysis to identify the key genes underlying the development of DR. Our study sought to elucidate the relationship between the circadian rhythm and DR, ultimately revealing novel key genes and potential mechanistic insights crucial for the management of this debilitating ocular condition.

## 2 Methods

### 2.1 Data acquisition

In this study, the dataset utilized for analyzing differential gene expression and conducting weighted gene co-expression network analysis (WGCNA) was obtained from the GEO database (https://www.ncbi.nlm.nih.gov/geo/). Specifically, the dataset GSE160306 was selected, comprising a collection of 20 normal samples and 39 samples from individuals diagnosed with DR. The Illumina HiSeq 4000 platform (Homo sapiens) was employed for sequencing the samples, ensuring high-quality data for subsequent analyses. To investigate the role of circadian rhythm in the context of DR, a comprehensive search was conducted within the Molecular Signatures Database (MsigDB) to identify genes associated with circadian rhythm (CRRS). The criteria used for gene selection involved excluding any duplicated or overlapping genes. As a result, a total of 300 circadian rhythm-related genes that met the defined criteria were successfully extracted from the MsigDB database. By integrating these curated circadian rhythm-related genes with the GSE160306 dataset, this study aimed to gain deeper insights into the relationship between circadian rhythm and DR. This approach holds promise for identifying novel key genes and uncovering potential underlying mechanisms that could pave the way for innovative treatment strategies targeting DR.

### 2.2 Differential expression analysis

To assess differential gene expression between normal samples and those affected by DR, differential expression analysis was performed using the R package ‘DESeq2’ (33). DESeq2 is based on the negative binomial distribution and offers a robust approach for analyzing count data obtained from high-throughput sequencing assays, leveraging the negative binomial distribution. By modeling the negative binomial distribution, DESeq2 effectively accounts for the inherent dispersion in gene expression levels across samples. Estimate variance-mean dependence in count data from high-throughput sequencing assays and test for differential expression based on a model using the negative binomial distribution. This approach enhances the reliability and validity of the results obtained.

### 2.3 Weighted gene co-expression network analysis

Weighted correlation network analysis (WGCNA) can be utilized to identify clusters (modules) of highly associated genes, summarize such clusters based on the module eigengene, and relate modules to external clinical traits (34). In brief, we preprocessed the raw gene expression profile data to remove low-quality reads, and correct for batch effects and technical variations between samples by quantifying expression levels, and normalizing the data. Then, a correlation or similarity matrix is constructed to represent gene relationships. Using a soft thresholding approach, highly co-expressed gene modules are identified. The modules are analyzed for preservation and significance. Hub genes with high connectivity within each module are identified. Functional enrichment analysis is performed to understand the biological processes associated with the modules and hub genes. Correlation networks can be used to screen candidate hub genes or therapeutic molecules.

### 2.4 LASSO analysis

Lasso regression is a type of linear regression that uses shrinkage. Shrinkage is where data values are shrunk toward a central point, like the mean. After preprocessing the input dataset, we choose the relevant features from the available independent variables to construct the model. Use domain knowledge, statistical methods, or machine learning algorithms to select features that have potential predictive power. And then the objective is to minimize the loss function, which is composed of a squared error term. The L1 regularization term penalizes the absolute values of the regression coefficients, promoting sparsity in the parameter set. The lasso procedure encourages simple, sparse models (i.e., models with fewer parameters). We interpret the model results based on the coefficients obtained, which indicate variable importance and positive/negative relationships with the target variable. This regression is well-suited for models showing high levels of muticollinearity or when you want to automate certain parts of model selection, like variable selection/parameter elimination.

### 2.5 Functional enrichment analysis

To conduct functional annotation of differentially expressed genes (DEGs), we utilized the R package “clusterProfiler” (version: 3.18.1) (35). This package offers a wide range of functional annotation tools that assist researchers in understanding the biological significance of specific gene sets. The clusterProfiler package relies on the Bioconductor annotation data GO.db and KEGG.db, which provide comprehensive maps for gene ontology (GO) analysis and the Kyoto Encyclopedia of Genes and Genomes (KEGG) corpus, respectively. We perform the differential expression analysis using appropriate statistical methods (edgeR or DESeq2) to obtain a list of DEGs based on defined criteria such as fold change and statistical significance. Then the clusterProfiler package was apply to annotate the identified DEGs. This may involve examining significantly enriched GO terms or KEGG pathways, and identifying biological processes or molecular functions that are overrepresented among the DEGs.

### 2.6 Single-sample gene set enrichment analysis

The single-sample gene set enrichment analysis (ssGSEA) was analyzed in our study by R package “GSVA” (36). In brief, the gene expression dataset was properly formatted and contains the necessary information for data preparation. The Gene Ontology (GO) terms, Kyoto Encyclopedia of Genes and Genomes (KEGG) pathways were selected for collection. The ssGSEA algorithm was applied from the GSVA package to each sample in the dataset. Interpretation of results involved identifying significantly enriched gene sets, assessing the variation of enrichment scores across samples, and exploring the biological implications of these findings. ssGSEA is a non-parametric, unsupervised method to calculate variation of gene set enrichment through the samples from an expression dataset.

### 2.7 Western blotting

The mice retinal tissues were subjected to milling and lysis using RIPA reagent (Beyotime, Shanghai, China) supplemented with a protease inhibitor, aiming to extract proteins. The isolated proteins were concentrated via the BCA method and then subjected to boiling at 95°C for 5 minutes. For vertical electrophoresis, 20 μg of protein was introduced into each well and separated by 12% SDS-PAGE at 110V for 1 hour. Subsequently, the proteins were transferred onto a PVDF membrane. The PVDF membrane was subsequently blocked using blocking serum for 1 hour before being incubated with primary antibodies, namely Anti-COL6A3 (1:1000; Abcam, Cambridge, MA, USA) and Anti-IGFBP2 (1:1000; Abcam, Cambridge, MA, USA), overnight at 4°C. The membrane was then exposed to a secondary antibody (1:1000; Abcam, Cambridge, USA) at room temperature for 1 hour. Finally, enhanced chemiluminescence reagent was added for protein band development, and the grayscale value of the protein bands was scanned using QUANTITY ONE software.

### 2.8 Reverse transcription-quantitative polymerase chain reaction (qRT-PCR)

Total RNA of mice retinal tissues was extracted using TRIzol solution (Invitrogen, Carlsbad, CA, USA). Subsequently, the RNA was reverse transcribed into cDNA using the Fast Quant RT Kit (TaKaRa, Otsu, Shiga, Japan). Quantitative real-time PCR (qPCR) was performed on an Applied Biosystems 7500 Real-Time PCR System (Thermo Fisher Scientific, Inc., Waltham, MA, USA), with SYBR Green Master Mix serving as the fluorogenic probe. The qPCR reaction protocol involved an initial preheating step at 95°C for 5 minutes, followed by denaturation at 95°C for 30 seconds, annealing at 60°C for 45 seconds for 40 cycles, and extension at 72°C for 30 minutes. For the amplification of COL6A3, the forward primer sequence used was 5’-AACATCCTGGTCAGCTCTGC-3’, and the reverse primer sequence was 5’-TCCGGGATGAAGGAGATGGT-3’. Similarly, the forward primer for IGFBP2 was 5’-CCTCAAGTCAGGCATGAAGGAG-3’, and the reverse primer was 5’-TGGTCCAACTCCTGCTGGCAAG-3’. Additionally, GAPDH was utilized as an internal control with the forward primer sequence 5’-CATCACTGCCACCCAGAAGACTG-3’ and the reverse primer sequence 5’-ATGCCAGTGAGCTTCCCGTTCAG-3’. All experiments were conducted in triplicate for validation. The relative expression levels were determined using the 2^^(-ΔΔCT)^ method, which allows for the quantification of relative expression levels when compared to internal control (GAPDH).

### 2.9 Animals

All animal procedures were conducted in strict accordance with the current national and international regulations governing animal care, housing, breeding, and experimentation. These protocols adhered to the guidelines set forth by the Association for Research in Vision and Ophthalmology (ARVO). The ethical committee (2021MER-041) approved all procedures involving animals. Wild-type male C57BL mice were housed in a temperature-controlled room (23 ± 1°C) with a 12-hour light-dark cycle, and they had ad libitum access to food and water. To induce diabetes, 3-week-old male mice were fasted and subjected to chemical induction as previously described (37–39). In brief, the animals received three consecutive intraperitoneal injections of STZ at a dosage of 85 mg/kg, dissolved in 0.01 M sodium citrate buffer (pH 4.5) during 3 successive days. Development of diabetes was weekly monitored via blood glucose levels. The induction of diabetes was confirmed by measuring blood glucose levels. Mice with blood glucose levels exceeding 2 g/L were considered diabetic. Only animals with consistent elevated blood glucose levels were considered diabetic and used in the study. Age-matched non-diabetic control mice received injections of 0.01 M sodium citrate buffer. The animals were maintained for a period of 10 weeks after the onset of diabetes for further experimentation. Mouse fundus tissue sections were prepared using frozen sectioning. After three 5-minute washes in PBS containing 0.3% Triton-X 100 (Solarbio, Beijing, China), the sections were blocked in a solution comprising 5% goat serum, 1% bovine serum albumin (Solarbio, Beijing, China), and 0.3% Triton-X 100 diluted in PBS (PBS-TX) for 1 hour at room temperature. Following this, the sections were separately incubated overnight at 4°C with primary antibodies to Collagen six. After three 10-minute washes in PBS-TX, the sections were then treated with Alexa Fluor 488-conjugated and Alexa Fluor 594-conjugated IgG (1:500, Abcam, Cambridge, UK), Phalloidin, and DAPI (1:500, Solarbio, Beijing, China) for 2 hours at room temperature, before undergoing three sequential 15-minute washes in PBS-TX. Subsequently, the slices were examined using laser scanning confocal microscopy (Zeiss LSM800; Zeiss, Oberkochen, Germany).

### 2.10 Statistical analysis

The statistical analysis of all data was performed using SPSS 22.0 software (SPSS Inc., Chicago, IL, USA) and GraphPad Prism 5.0 (GraphPad Software, San Diego, CA, USA). The results were presented as mean ± standard deviation (SD). A comparative analysis between the two groups was carried out using the student’s t-test. A significance level of *p* < 0.05 was considered statistically significant.

## 3 Results

### 3.1 Differential expression analysis and clustering reveals distinct expression patterns in diabetic retinopathy samples

**Figure 1:**
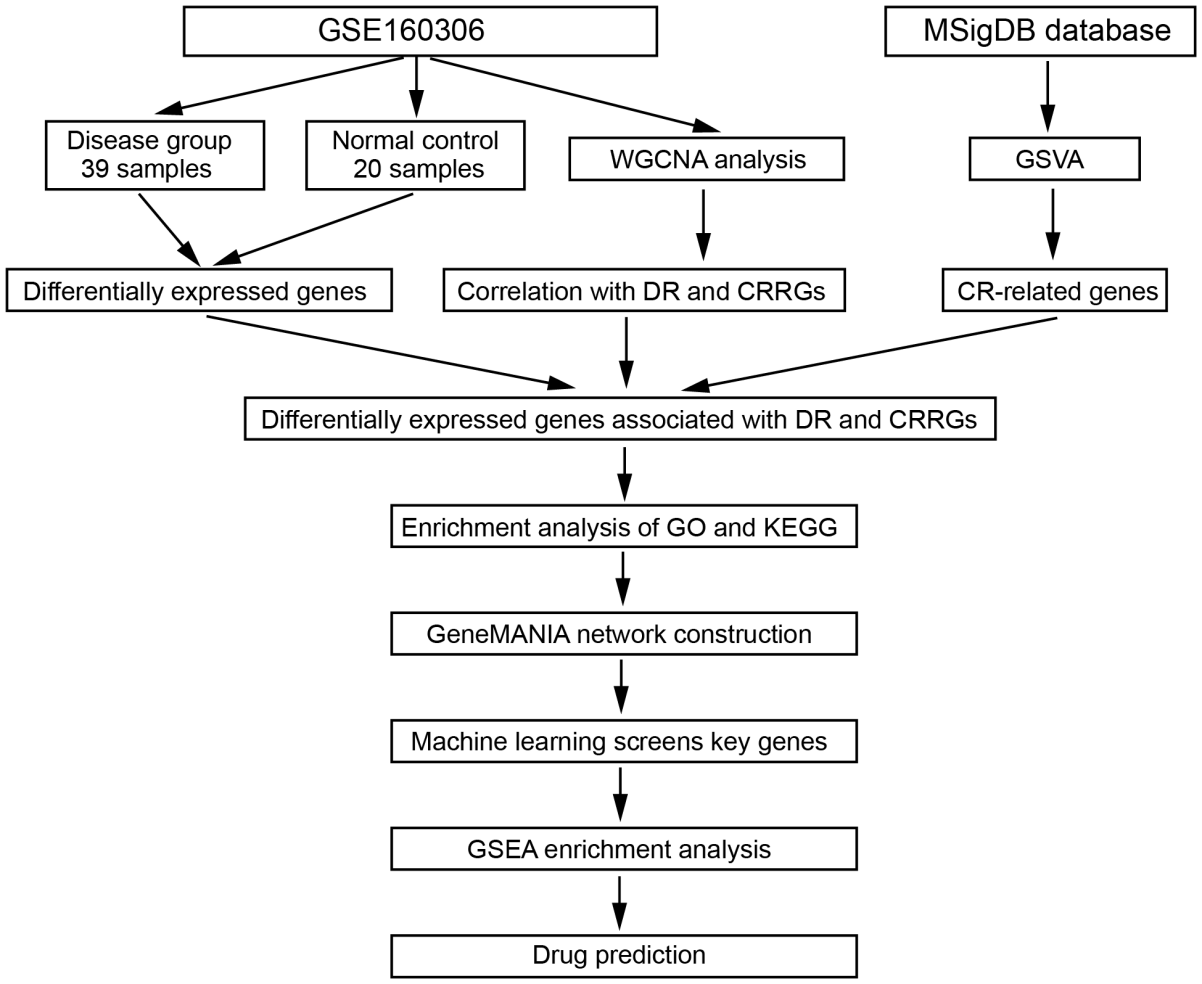
The workflow of this study.

**Figure 2:**
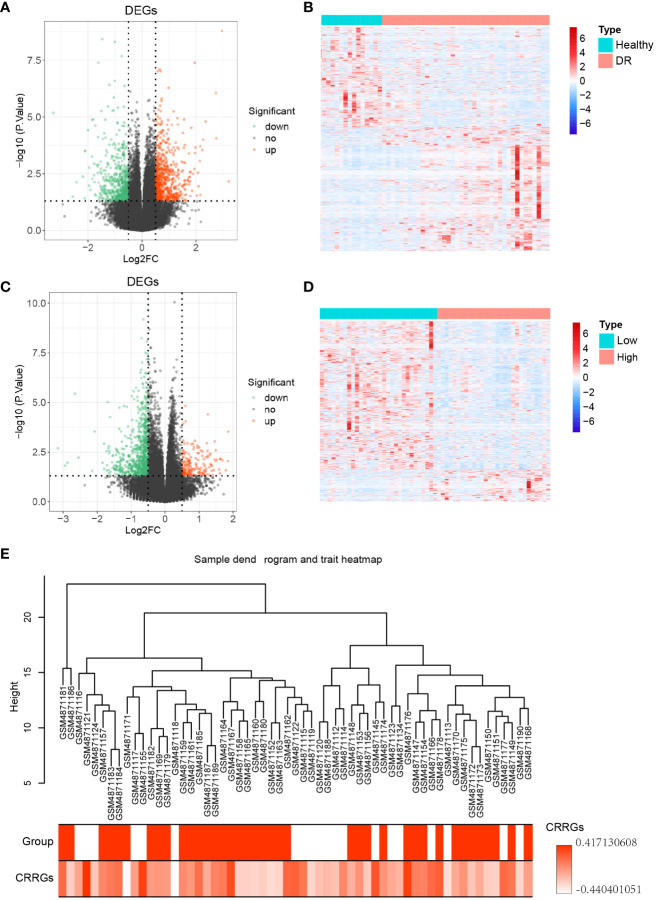
Differential genes expression analysis. **(A)** Volcano plot illustrating the differentially expressed genes (DEGs) between the DR and normal samples. **(B)** Heatmap displaying the expression levels of the DEGs between the DR and normal samples. **(C)** Volcano plot illustrating the DEGs between the low and high groups. **(D)** Heatmap displaying the expression levels of the DEGs between the low and high groups. **(E)** Sample dendrogram and trait heatmap representing 59 samples.

To identify differentially expressed genes (DEGs) between DR and normal samples, a differential expression analysis was conducted on 20 normal samples and 39 DR samples from the GSE160306 dataset using the R package ‘DESeq2’ (**Figure 1**). The analysis revealed a total of 1253 DEGs, consisting of 690 upregulated genes and 563 downregulated genes (p < 0.05, |log FC| > 0.05; **Figure 2A**). To further explore the distribution of DEGs between the normal and DR groups, a heatmap displaying the expression levels of the DEGs was generated (**Figure 2B**), highlighting distinct expression patterns between the two groups.

Subsequently, the expression levels of the circadian rhythm were quantified for each sample using the GSVA algorithm, based on the circadian rhythm gene set. The samples were divided into low and high groups according to the median value of the circadian rhythm expression levels. Differential expression analysis was then performed between these two groups using the R package ‘DESeq2’, resulting in 935 DEGs. Among these DEGs, 167 were upregulated genes and 768 were downregulated genes (p < 0.05, |log FC| > 0.05; **Figure 2C**). To assess the distribution of DEGs between the low and high groups, a heatmap displaying the expression levels of the DEGs was generated (**Figure 2D**), revealing distinct expression patterns between the two groups. Furthermore, a clustering analysis was conducted on the 59 samples in the GSE160306 dataset to identify potential outlier samples. The analysis did not reveal any outlier samples within the sample dendrogram (**Figure 2E**).

### 3.2 Deciphering the interplay between CR and DR by weighting gene co-expression network analysis

**Figure 3:**
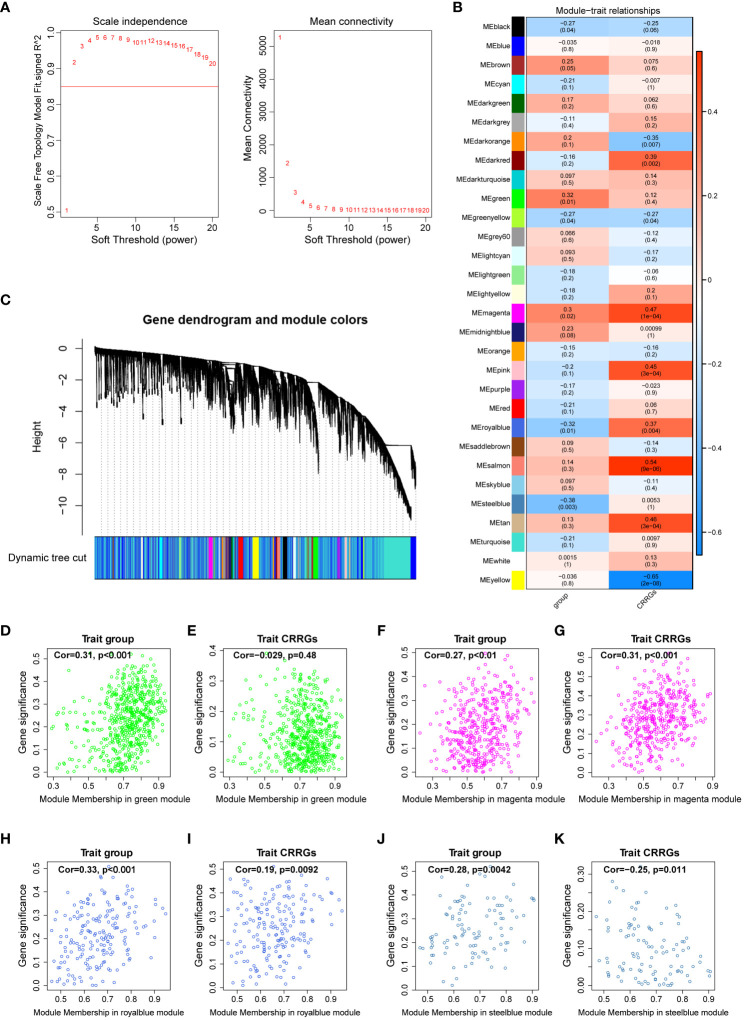
Weighted gene co-expression network analysis. **(A)** Determination of the optimal soft threshold utilized for gene clustering. **(B)** Analysis of the relationships between modules and traits, including group and circadian rhythm (CR). **(C)** Dendrogram of all differentially expressed genes from the dynamic tree cut. **(D, E)** Dot plots illustrating the distribution of genes within the green module. **(F, G)** Dot plots illustrating the distribution of genes within the magenta module. **(H, I)** Dot plots illustrating the distribution of genes within the royalblue module. **(J, K)** Dot plots illustrating the distribution of genes within the steelblue module.

To elucidate the gene modules relevant to the circadian rhythm (CR) trait, we conducted a weighted gene co-expression network analysis (WGCNA) on a dataset comprising 59 samples. Firstly, we determined the appropriate soft threshold to enhance gene clustering. By assessing the y-intercept of the red line, we identified 2 as the optimal soft threshold (**Figure 3A**). Subsequently, employing the determined soft threshold, we partitioned the genes into 30 distinct modules utilizing the dynamic tree cutting algorithm. We proceeded to analyze the correlation between these modules and the traits of interest (**Figures 3B, C**). Remarkably, we identified four modules strongly associated with both the DR and CR traits, namely the green, magenta, royalblue, and steelblue modules (**Figures 3D–K**). These four modules were subsequently recognized as the key gene modules underlying the complex interplay between DR and CR. The identification of these key gene modules provides vital insights into the molecular mechanisms governing the pathogenesis of diabetic retinopathy and its relationship with the circadian rhythm.

### 3.3 Unraveling circadian rhythm-related genes in diabetic retinopathy

**Figure 4:**
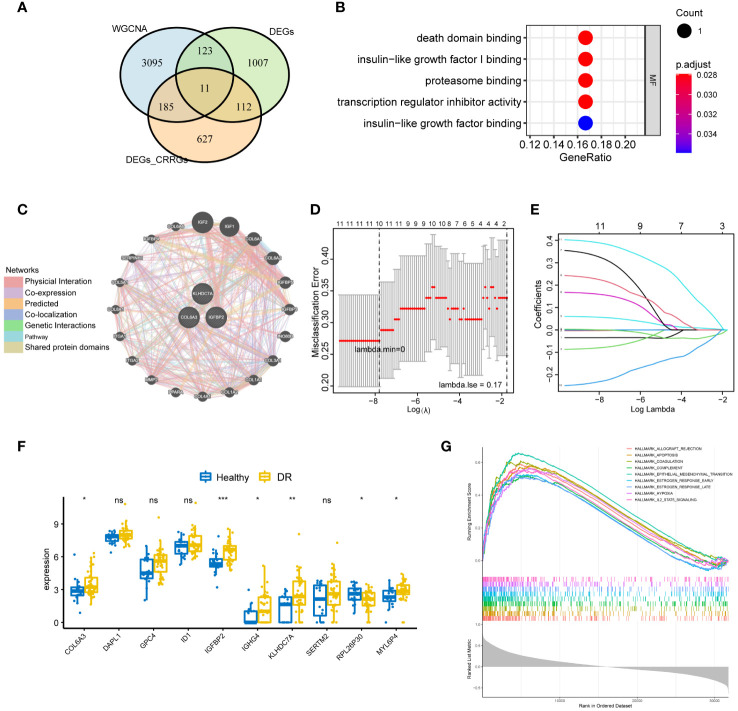
Identification of key CRRGs. **(A)** 11 overlapping genes were obtained by intersecting the genes in WGCNA, DEGs between the normal and DR groups, as well as that between the low and high group. **(B)** Functional enrichment analysis illuminating the signaling pathways associated with the identified 11 overlapping CRRGs. **(C)** Interaction network showcasing the connections between the 11 CRRGs and 20 related genes, as revealed by analysis using the GeneMANIA database. **(D, E)** LASSO regression analysis for further refinement, leading to the retention of 10 key genes among the 11 CRRGs. **(F)** Box plots visually representing the differential expression levels of the 10 genes identified through LASSO regression, thereby differentiating between the DR and healthy groups. **(G)** GSEA highlighting the enriched pathways related to allograft rejection, apoptosis, coagulation, and hypoxia for the 10 genes obtained through LASSO regression. n = 6. Data are presented as mean ± SD. ns, no statistical. *p < 0.05, **p < 0.01, and ***p < 0.001 between the lined group.

To identify crucial circadian rhythm-related genes (CRRGs) in DR, we performed an intersection analysis among the genes identified in the WGCNA, the differentially expressed genes between the normal and DR groups, as well as the DEGs between the low and high group. This analysis yielded 11 overlapping genes, namely *COL6A3*, *DAPL1*, *GPC4*, *ID1*, *IGFBP2*, *IGHG4*, *KLHDC7A*, *SERTM2*, *LINC02679*, *RPL26P30*, and *MYL6P4* (**Figure 4A**). Next, we conducted functional enrichment analysis to investigate the biological implications of these 11 genes. This analysis revealed enrichment for several signaling pathways, including death domain binding, insulin-like growth factor I binding, and proteasome binding (**Figure 4B**).

**Figure 5:**
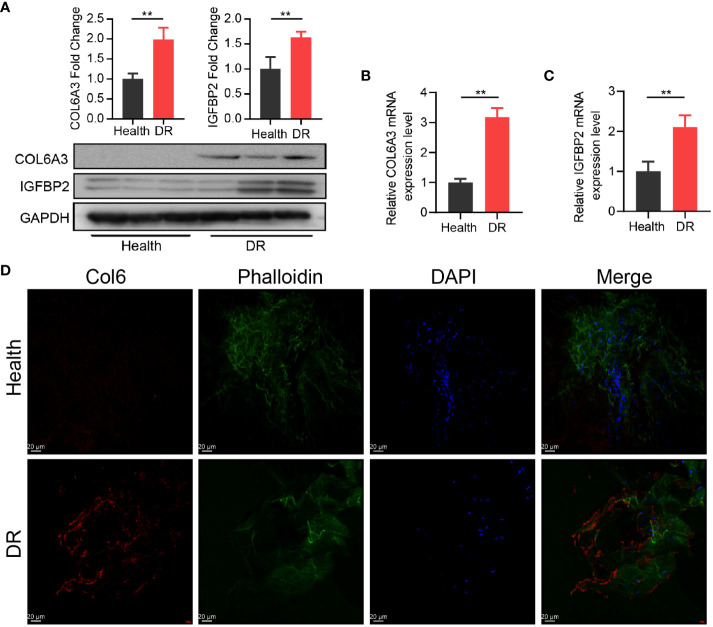
High expression of CR-related genes was associated with DR. **(A)** Relative expression of COL6A3 between health tissue and DR tissue from the diabetes model. **(B, C)** Relative expression of COL6A3 and IGFBP2 between health tissue and DR tissue were analysised by qRT-PCR. **(D)** Images of Immunofluorescence staining of retinal tissue for COL6A3 (red). Cell nuclei were counterstained with DAPI (blue) and actin filaments with Phalloidin (green). Scale bar: 20 µm. n = 5 biologically independent animals. Data are presented as mean ± SD. ns: no statistical. **p < 0.01 between the lined group.

Furthermore, we explored the interaction between the 11 CRRGs and 20 related genes using the GeneMANIA database. The resulting interaction network consisted of 23 nodes and 793 edges (**Figure 4C**). To further refine the selection of key genes involved in the progression of DR, we employed LASSO regression analysis on the 11 CRRGs. As a result, 10 genes were retained (**Figures 4D, E**). Box plots were generated to visualize the expression levels of these 10 genes, revealing that six genes exhibited significant differential expression between the normal and DR groups. Hence, we identified *COL6A3*, *IGFBP2*, *IGHG4*, *KLHDC7A*, *RPL26P30*, and *MYL6P4* as the final key genes (**Figure 4F**). Subsequent Gene Set Enrichment Analysis (GSEA) demonstrated that these six genes were enriched in pathways related to *allograft rejection*, *apoptosis*, *coagulation*, and *hypoxia* (**Figure 4G**). To validate our findings, we collected tissue samples from healthy individuals and DR models to examine the expression levels of COL6A3 and IGFB2. Remarkably, compared to normal tissue, the expression levels of *COL6A3* and IGFB2 were significantly increased in DR tissue (**Figures 5A–D**). Collectively, these findings offer compelling evidence, strengthening the association between the development of DR and the aberrant expression of COL6A3 and IGFB2.

### 3.4 Investigation of the effect of key genes in DR

**Figure 6:**
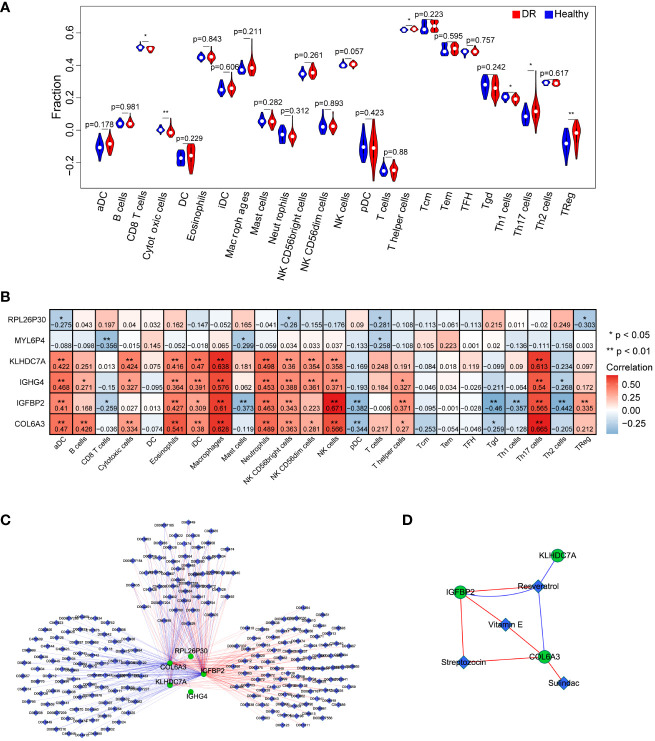
Investigation of the effect of key genes in immunomodulation and drug targets. **(A)** Comparison of immune cells between the DR and healthy group. **(B)** The correlation analysis between key genes and immune cells. **(C)** the interaction network of the six key genes and the relative agents related to the key genes. **(D)** Four drugs were obtained that were associated with key genes, including Resveratrol, Vitamin E, Streptozocin, and Sulindac. n = 6. Data are presented as mean ± SD. ns, no statistical. *p < 0.05 and **p < 0.01 between the lined group.

To investigate the immune microenvironment of diabetic retinopathy (DR), we conducted an analysis of immune cells in DR samples. Initially, we employed the single-sample gene set enrichment analysis (ssGSEA) algorithm to quantify the relative scores of 24 immune cell types in individual samples. Comparing these scores between the healthy and DR groups, we identified six immune cell types that exhibited significant differences: CD8 T cells, cytotoxic cells, T helper cells, Th1 cells, Th17 cells, and TReg cells (**Figure 6A**). Furthermore, we examined the relationship between these six hub genes and the aforementioned immune cell types. Our analysis revealed that COL6A3 displayed a positive correlation with macrophages (cor=0.628296895, p=9.96E-08) and Th17 cells (cor=0.665120835, p=9.14E-09). Conversely, IGFBP2 exhibited a negative correlation with Tgd (cor=-0.459953045, p=0.000247284) and Th2 cells (cor=-0.442269719, p=0.000452875), as showed in **Figure 6B**.

To explore potential therapeutic agents related to the key genes, we consulted the Comparative Toxicogenomics Database (CTD) (http://ctdbase.org/) and extracted relevant drug or agent information. Subsequently, we visualized the interaction network among these molecules using Cytoscape. **Figure 6C** illustrates a network comprising 228 nodes and 353 edges, consisting of 179 stimulatory relationships and 174 inhibitory relationships. Notably, we identified four drugs that were associated with the targeted key genes: Resveratrol, Vitamin E, Streptozocin, and Sulindac (**Figure 6D**). Overall, these findings contribute to a better understanding of the immune dysregulation in DR and provide insights into potential therapeutic avenues for this condition.

## 4 Discussion

In the present study, we sought to elucidate the presence of the CR-related genes in DR. Notably, we have successfully identified six pivotal CR-related genes that hold potential therapeutic value for the treatment of DR, paving the way for innovative therapeutic interventions. Our meticulous analysis has also brought to the fore enriched signaling pathways linked to death domain binding, insulin-like growth factor I binding, and proteasome binding. Furthermore, we have unveiled an intriguing correlation between CR-related genes and various immune cell populations within the DR microenvironment. Collectively, these significant findings shed light on the identification of novel pathogenic genes implicated in DR, thereby laying the foundation for the development of precision medicine approaches.

Multiple studies have provided evidence supporting the regulatory role of retinal ganglion cells, which contain the photopigment melanopsin, in modulating the circadian clock. Additionally, retinal changes associated with blindness have been shown to impact circadian rhythms (40–42). In line with these previous findings, our study offers a novel contribution by identifying CR-related genes with potential therapeutic applications in the treatment of DR. The identified genes include *COL6A3*, *IGFBP2*, *IGHG4*, *KLHDC7A*, and *RPL26P30*. Notably, previous research has reported upregulation of *COL6A3* and *KLHDC7A* in DR, which is consistent with our findings (43, 44).. Although there is currently no reported association between DR and *IGFBP2*, *IGHG4*, *RPL26P30*, as well as *MYL6P4*, the novel nature of their identification warrants further investigation. The delineation of these six pivotal CR-related genes and their potential therapeutic implications in the context of DR emphasizes the translational significance of our study. Furthermore, these findings not only enhance understanding of the underlying molecular processes driving DR pathogenesis but also present promising targets for therapeutic intervention. Future experimental studies should focus on elucidating the precise mechanisms through which these genes exert their effects (45). Such investigations will provide additional insights into their functional roles and aid in advancing our understanding of the complex mechanisms underlying DR.

Another significant finding of our study was the identification of several key biological processes implicated in diabetic retinopathy (DR) and its high-risk state. These processes include death domain binding, insulin-like growth factor I binding, and proteasome binding (46). Notably, death domain binding emerges as a crucial factor in DR pathogenesis. Pyroptosis, recognized as a form of programmed-inflammatory cell death, has recently been associated with death domain binding and has shown promise in the treatment of DR (47). Furthermore, the role of insulin-like growth factor I binding as a pivotal factor in type 1 diabetic retinopathy has already been acknowledged in previous studies (48–50). We successfully revealed these important signaling pathways in DR, which would add novel knowledge to the current understanding of DR. The enrichment of specific signaling pathways, including death domain binding, insulin-like growth factor I binding, and proteasome binding, provides invaluable knowledge regarding the molecular mechanisms underlying the progression of DR (51). These findings not only advance our understanding of the disease but also open potential avenues for the development of precision medicine strategies targeting circadian regulation in the management and treatment of diabetic retinopathy.

Through an intersection analysis of CRRGs identified through WGCNA, DEGs between normal and DR groups, and DEGs between low and high-risk groups, we successfully identified 11 overlapping genes. Functional enrichment analysis unveiled the involvement of these genes in diverse signaling pathways, shedding light on their functional significance. Moreover, network analysis revealed potential interactions between the CRRGs and related genes, enhancing our understanding of the underlying molecular processes. To refine our findings and identify key genes, we applied LASSO regression, which led us to narrow down to 10 genes. Remarkably, six of these genes exhibited significant differential expression between normal and DR groups, reinforcing their potential role in DR pathogenesis. GSEA further validated the functional relevance of these genes, consolidating their importance. Furthermore, in our pursuit to explore potential therapeutic agents associated with these key genes, we uncovered Resveratrol, Vitamin E, Streptozocin, and Sulindac as promising candidates.

Vascular endothelial growth factor (VEGF) plays a central role in driving the process of vascular proliferation in proliferative diabetic retinopathy. Anti-VEGF drugs targeting VEGF have been extensively studied in diabetic macular edema and shown promising clinical efficacy (52). encodes a binding protein for insulin-like growth factor 1 (IGF1), which is known to interact with VEGF signaling pathways. Numerous studies have demonstrated the crucial role of IGFBP2 in promoting pathologic angiogenesis in various cancer types (53, 54). *IGFBP2* is considered an inducer of angiogenesis in melanoma. *IGFBP2* is believed to induce angiogenesis in melanoma by up-regulating the expression of pro-angiogenic VEGF-A, and subsequently triggering angiogenesis via interacting with integrin αVβ3 and activating the PI3K/AKT signaling cascade (55). Therefore, IGFBP2 may influence neovascularization in diabetic retinopathy through modulating VEGF activity mediated by IGF1. Additionally, *IGHG4* encodes an immunoglobulin protein involved in immune cell trafficking and inflammation. Chronic inflammation has been linked to angiogenesis, suggesting IGHG4 could contribute to neovascularization in proliferative diabetic retinopathy through inflammation (56). However, further investigation is warranted to ascertain their efficacy and safety profiles specifically in the context of DR treatment. Considering the sight-threatening nature of DR treatment, many preventive and therapeutic modalities have been proposed. Thus, future research directions should focus on evaluating the efficacy of these agents in preclinical and clinical settings, elucidating their underlying mechanisms of action, and exploring potential combination therapies. These findings contribute to a better understanding of the immune dysregulation in DR and provide insights into potential therapeutic avenues for this condition.

The mechanisms underlying leukocyte activation in diabetes remain incompletely defined. Recent studies have demonstrated elevated levels of phosphorylated signal transducer and activator of transcription 3 (pSTAT3), a key regulatory molecule in cell signaling, in circulating myeloid (monocyte/macrophage) cells from patients with diabetes and in STZ-induced diabetic mice (57).. Additionally, patients with DR have shown increased levels of various autoantibodies, including hexokinase 1. Interestingly, one study found a higher prevalence of DR in the absence of autoantibodies (58, 59).. Moreover, a prospective pilot study revealed an increased number of circulating neutrophilic leukocytes and a reduced number of T cells during the development and progression of DR (60). One intriguing aspect of our investigation is the observed association between the co-expressed retina-related genes (CR-related genes) and different immune cell populations within the microenvironment of DR. Our analysis of the immune microenvironment in DR unveiled significant alterations in immune cell populations compared to healthy tissue. Specifically, CD8 T cells, cytotoxic cells, T helper cells, Th1 cells, Th17 cells, and Treg cells exhibited marked differences between DR and healthy samples. It is important to note that further mechanistic studies are necessary to fully elucidate the precise roles of these immune cell types and their interactions with COL6A3 and IGFBP2 in the development and progression of diabetic retinopathy. In addition, we identified a positive correlation between the expression of COL6A3 and the presence of macrophages and Th17 cells Conversely, IGFBP2 demonstrated a negative correlation with Tgd and Th2 cells (61). Overall, our findings provide a solid foundation for future research in the field of diabetic retinopathy immunopathology and the development of therapeutic interventions.

The current study presents certain limitations, highlighting the need for further investigation. First, the pivotal genes and signaling pathways in the present study were obtained by bioinformatics approaches, and lab experiments are needed to investigate and validate their specific roles. Also, the drugs were identified based on the online database without drug response experiment, requiring further pre-clinical investigation. Our analysis utilized the ssGSEA algorithm to quantify immune cell scores. It is important to note that alternative methods could be employed to validate and complement these findings. For instance, Single-cell RNA sequencing (scRNA-seq) approaches could provide a more detailed and comprehensive understanding of the immune cell landscape in DR, enabling the identification of additional cell subsets and providing a more in-depth understanding of their functional diversity.

## 5 Conclusion

In summary, our integrative analysis, combining differential expression and co-expression analyses, has revealed six novel CR-related genes and their corresponding drugs as potential therapeutic options for DR. This study provides a fresh perspective on DR pathogenesis, complementing existing knowledge and offering future directions for precision medicine in this domain. The integration of these novel therapeutic targets and the delineation of immune cell interactions hold promise for the advancement of individualized treatment strategies to tackle the complexities of DR.

## Data availability statement

The original contributions presented in the study are included in the article/supplementary material. Further inquiries can be directed to the corresponding author.

## Ethics statement

The animal study was approved by Inner Mongolia Baogang Hospital ethics committee review meeting. The study was conducted in accordance with the local legislation and institutional requirements.

## Author contributions

Conceptualization: SZ. Methodology and software: FL and CZ. Visualization: CZ and XX. Writing: FL. Review and editing: SZ. Supervision and acquisition of funding: XZ. All authors contributed to the article and approved the submitted version.
